# Analysis of the Behaviours Mediating Barnacle Cyprid Reversible Adhesion

**DOI:** 10.1371/journal.pone.0068085

**Published:** 2013-07-11

**Authors:** Nick Aldred, Jens T. Høeg, Diego Maruzzo, Anthony S. Clare

**Affiliations:** 1 School of Marine Science and Technology, Newcastle University, Newcastle upon Tyne, United Kingdom; 2 Department of Biology, University of Copenhagen, Copenhagen, Denmark; 3 Department of Biology, University of Padova, Padova, Italy; University of Zurich, Switzerland

## Abstract

When exploring immersed surfaces the cypris larvae of barnacles employ a tenacious and rapidly reversible adhesion mechanism to facilitate their characteristic ‘walking’ behaviour. Although of direct relevance to the fields of marine biofouling and bio-inspired adhesive development, the mechanism of temporary adhesion in cyprids remains poorly understood. Cyprids secrete deposits of a proteinaceous substance during surface attachment and these are often visible as ‘footprints’ on previously explored surfaces. The attachment structures, the antennular discs, of cyprids also present a complex morphology reminiscent of both the hairy appendages used by some terrestrial invertebrates for temporary adhesion and a classic ‘suction cup’. Despite the numerous analytical approaches so-far employed, it has not been possible to resolve conclusively the respective contributions of viscoelastic adhesion via the proteinaceous ‘temporary adhesive’, ‘dry’ adhesion via the cuticular villi present on the disc and the behavioural contribution by the organism. In this study, high-speed photography was used for the first time to capture the behaviour of cyprids at the instant of temporary attachment and detachment. Attachment is facilitated by a constantly sticky disc surface – presumably due to the presence of the proteinaceous temporary adhesive. The tenacity of the resulting bond, however, is mediated behaviourally. For weak attachment the disc is constantly moved on the surface, whereas for a strong attachment the disc is spread out on the surface. Voluntary detachment is by force, requiring twisting or peeling of the bond – seemingly without any more subtle detachment behaviours. Micro-bubbles were observed at the adhesive interface as the cyprid detached, possibly an adaptation for energy dissipation. These observations will allow future work to focus more specifically on the cyprid temporary adhesive proteins, which appear to be fundamental to adhesion, inherently sticky and exquisitely adapted for reversible adhesion underwater.

## Introduction

**Figure 1:**
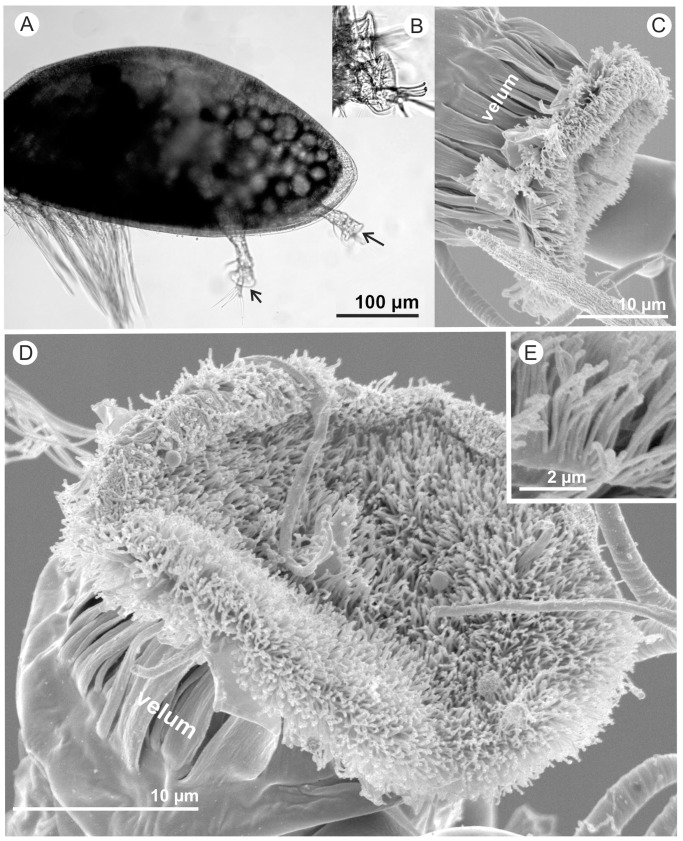
A cyprid of *Semibalanus balanoides.* A) A low power light micrograph of a cyprid with frontal antennules and thoracic swimming appendages visible; arrows point to the third segments bearing the antennular discs. B) Detail of antennular discs used for adhesion. C) SEM; near medial view of the third antennular segment; narrow velar flaps encircle the third segment reaching to the periphery of the antennular disc. D) Face-on-view of the villus-covered antennular disc. E) Detail from the rim of the attachment disc where velar flaps were missing, allowing estimation of length of the villi.

Cyprids are the final larval stage of barnacles and are superbly equipped for surface exploration, selection of a suitable location for settlement and final attachment prior to metamorphosis to the adult form [1]–[2]. Using adhesive discs on the 3rd segments of paired antennules, cyprids are able to walk in a rapidly reversible bi-pedal fashion over immersed surfaces [3] (Figure 1), evaluating the properties of the substratum using a range of sensory structures [4]. Most of the cyprid's external sensory setae are concentrated on the 3rd and 4th segments of the antennules, including on and around the adhesive disc, with putative chemo-, contact-chemo- and mechano-sensory setae having been described in detail [4]–[6]. Although specific details of the antennule-associated structures differ between barnacle species, perhaps corresponding to preferred habitat type [5], the gross morphology of the antennules and the sensory and adhesive structures they support are universally conserved. Only relatively minor differences in cyprid morphology [7] have been identified between species with vastly different adult forms (e.g. balanid, lepadid and sacculinid barnacles). The similarity of adhesive structures between species, regardless of adult form, is suggestive of a supremely adapted adhesion mechanism.

The cyprid is of particular interest in the context of marine biofouling, due to the economic implications of settlement and growth of barnacles on anthropogenic structures [8]–[9]. Additionally, the tenacious and rapidly-reversible underwater adhesion mechanism used by cyprids during surface exploration is unique among aquatic invertebrates and may also serve as a potential model for the development of synthetic, bio-inspired temporary adhesion systems (e.g. [10]). One approach towards reducing the fouling impact of barnacles is to develop surfaces to which temporary and/or permanent adhesion by the cyprid is impossible (e.g. [11]–[12]). So far, however, progress in this field has been restricted by a poor understanding of the mechanisms of adhesion used by cyprids, both temporary and permanent, during attachment to surfaces.

Historically, quantitative studies of cyprid temporary adhesion have been prohibitively difficult and/or time-consuming to perform, due to the micron-scale and great speed of the behaviours. This lack of empirical evidence has contributed to decades of speculation as to the possible mechanism of temporary adhesion, with most hypotheses informed only by proxy evidence such as adhesion strength to various substrata [13]–[14] and imaging of adhesive deposits on surfaces [15]–[22].

For many years the ability of cyprids to rapidly and tenaciously attach to surfaces underwater was assigned to suction developed beneath the adhesive discs at the tips of the paired antennules [23]. Nott and Foster [24] first rejected the suction hypothesis on the basis of morphology; cyprids simply do not have the musculature required to develop a self-contained low-pressure environment beneath the antennular discs ([25]. The argument put forward by Nott and Foster was later supported by Crisp [26], who cited observations by his laboratory members that cyprids of *Semibalanus balanoides* can attach to surfaces narrower in diameter than the adhesive disc. Nevertheless, references to suction persisted in the literature until at least 1984 [23] and it was not until experimental evidence was presented on the tenacity of the larvae to various surfaces [14] and the internal structure of the third antennular segment was elucidated [25] that the suction theory was finally refuted. The only muscle distal to the second antennular segment is a single one that operates the flexing movements of the cylindrical fourth segment [24] and there are no muscles that could move the attachment disc cuticle in such a way as to invoke suction in any cirripede cyprid. Adhesion strength of temporarily attached cyprids was also shown to vary according to the chemical composition of the substratum [13]–[14] and suction would provide the same adhesion strength for all smooth surfaces, irrespective of their chemical character.

The alternative hypothesis advanced to explain the mechanism of reversible adhesion was the use of an adhesive. Walker and Yule [15] were the first to demonstrate involvement of a liquid secretion in cyprid reversible adhesion – a material they termed “cyprid temporary adhesive”. Footprints of this substance were identified on silanized glass surfaces after exploration by cyprids. Using scanning electron microscopy, Walker and Yule estimated the thickness of these deposits to be on the order of 15 nm - an estimate remarkably close to more recent independent values obtained by atomic force microscopy [18] and imaging surface plasmon resonance [21]–[22]. Although identifying the existence of this adhesive secretion was a significant step forward it did not, in itself, adequately explain the mechanism of rapid attachment and, more specifically, detachment in exploring cyprids. The debate was further confounded by the emerging possibility that the secretion may act as a conspecific settlement cue to other exploring cyprids [16], [27] rather than exclusively as an adhesive. The component of the temporary adhesive that is active in conspecific signalling is the settlement-inducing protein complex (SIPC) present in larval and adult barnacles [17], [28], thus raising the possibility that in cyprid temporary adhesion the SIPC may serve a dual role, as an adhesive molecule as well as a conspecific pheromone [29].

With few techniques available to characterise interfacial adhesive processes at and below the microscale, mechanistic explanations for cyprid temporary adhesion were hypothetical in nature. Theories included a ‘duo-gland’ adhesive system (discussed in [26]), similar to that used by echinoderms for temporary attachment to surfaces [30], and so-called Stéfan adhesion [26]. Using atomic force microscopy, Phang et al. [18] described the morphology of deposited cyprid footprints in greater detail and preferred the terminology “footprint material” to describe the temporary adhesive, given the continuing ambiguity as to its exact role. Although they found no direct evidence for a mechanism of action, Phang et al. [18] proposed the possibility that the cuticular villi or ‘hairs’ covering the attachment disc may be involved in adhesion to surfaces (first proposed by Crisp [26] in a similar way to the cuticular hairs covering the pulvilli of dipteran flies [9], [31]. Phang et al. [20] also identified common characteristics of adhesive proteins in the cyprid footprint material, such as intermolecular sacrificial bonds, supporting the involvement of this material in temporary adhesion. Despite these studies and several other attempts to use advanced biophysical methods to determine the relative adhesive contributions of the footprint material, the morphology of the disc and the behaviour of the cyprid during adhesion, the entire process of temporary adhesion remains poorly understood.

In this study, we use a combination of high magnification light microscopy and high-speed photography to provide insight into the mechanism of temporary adhesion in cyprids. Although the resolution of the high-speed image acquisition was incomparable to techniques such as scanning electron microscopy or high-resolution light microscopy used for fine morphological descriptions, the method presented significant advantages and leads the way for more penetrating biomechanical experimentation. The phenomena described in this paper were observed many times, during many hours of direct observation of cyprids, although due to the speed of movement of the organisms, ephemeral nature of the specific behaviour under investigation, negligible depth of field and restricted field of view inherant in the technique, only occasionally were high-quality recordings captured for further analysis. Nevertheless, this approach revealed subtleties of behaviour that go a long way towards finally elucidating the mechanism of cyprid temporary adhesion. The recordings also highlight several previously unreported adaptations that are believed to maximise adhesion strength and minimise involuntary removal of cyprids during surface exploration.

## Methods

### Collection of Cyprids

Cyprids of the acorn barnacle *Semibalanus balanoides* were collected by plankton tow from the harbour at the Dove Marine Laboratory, Cullercoats, North East England in April 2010. Specific permission was not required for small sample collection. The cyprids were separated from other planktonic organisms and stored in 2-L glass beakers containing natural seawater (FSW), filtered using 0.2 µm nitrocellulose filter membranes (Millipore). Cyprids were stored until use at 6°C in the dark. Microscopy was carried out at the Department of Biology, University of Copenhagen, where cyprids were sent in advance using an overnight courier service and then stored as previously.

### Video Microscopy

Cyprids were observed in the cells of modified BD Falcon 8-chamber Culture Slides (BD Biosciences No. 354108). All surfaces presented to the cyprids were clean, unmodified polystyrene. Each cell contained 200 µl of FSW and one *S. balanoides* cyprid. The cyprids were followed manually using an Olympus IX-70 inverted microscope with a 60x PLAN objective lens. Behaviours associated with surface exploration/temporary adhesion were recorded using an Olympus i-Speed 1 GB high-speed digital camera with 10× magnification via c-mount, and all video capture was carried out at 500 frames per second. In this way, cyprid exploration could be observed on the base and up the walls of the chamber slides, providing an inverted image and side profile respectively. Chamber slides were swapped frequently (around every 5-minutes) due to the warming effect of the microscope lamp and rested on cold-storage blocks between observations. Cyprids in the chambers were changed every hour.

Unless otherwise stated, all observations pertain to cyprids engaging in normal exploratory behaviour within the cells without intervention by the experimenter. To observe the behaviour of the temporary adhesive system under applied force, the water in the cell of interest was triturated using a Pasteur pipette, leading often to dislodgement of the cyprid.

Due to the speed of the movements and high magnification required to observe the features of interest, only rarely were the desired instants of attachment/detachment captured in high-quality movies. The movies uploaded here as supplementary electronic material are representative of the behaviours observed. The present description is, however, augmented by observations of behaviours that were not successfully captured. To allow quantitative description of some effects, calibrated measurements were made from stills taken from the captured movies in ImageJ software. Mean values and standard deviations were then calculated manually.

### Scanning Electron Microscopy

To estimate the density of villi on the antennular discs, cyprids were processed for SEM as explained in [5]. Three cyprids with a total of five antennular discs exposed to view were selected for close study. Face-on micrographs were taken with with a JEOL JSM-6335 SEM at low magnification to estimate the disc area. The area was calculated by considering the disc as an oval and measuring its long and short axes. The overviews also showed that the density of villi appeared to be homogenous over the entire disc area. From each disc 3–4 different areas were selected for counting density of villi. Each of these was photographed at 7,500x by carefully tilting the specimen so a near vertical view of the disc surface was obtained. The number of villi within a 3×3 µm^2^ area was then counted manually from print outs of the micrographs.

## Results and Discussion

### Voluntary Attachment and Detachment from Surfaces

During a normal ‘wide search’ stride (*sensu* Crisp [32]), contact between the adhesive disc of the lead antennule and the surface occurs first at the rear of the disc and ends with detachment at the front of the same disc on completion of the stride (Movies S1 & S2). Full contact between the disc and the surface is very brief during wide searching, similar in essence to the contact between a foot and a surface during a human stride. Usually, less than 50% of the adhesive disc is in contact with the surface. If the cyprid stops exploring and remains stationary for longer than a second or so, the adhesive disc is constantly ‘rolled’ on the surface (Movie S3) minimising the contact time between any one part of the disc and the surface. It was clear, therefore, that momentary contact of the adhesive disc to the surface yields attachment, but not strong attachment, and that minimisation of contact time/contact area is probably an adaptation to allow rapid walking with bare-minimum removal force required between steps. This behaviour was especially evident during ‘inspection’ behaviour [25]–[32], where one antennule makes intermittent contact with the substratum while the other maintains a more secure pivot point. The more secure, but still temporary, attachment used as a pivot point is achieved by allowing total contact of the disc to the surface for several seconds. The disc is pressed onto the surface, spreading it out and increasing the contact area by 24.9% on average (±4.9 SD, n = 4). The absence of this spreading in excised antennules may therefore explain the inability of Yule and Crisp [13] to measure attachment strength as they could for live cyprids. Strong temporary adhesion was concluded to be a behaviour-mediated phenomenon, facilitated by the naturally ‘sticky’ surface of the antennular disc.

**Figure 2:**
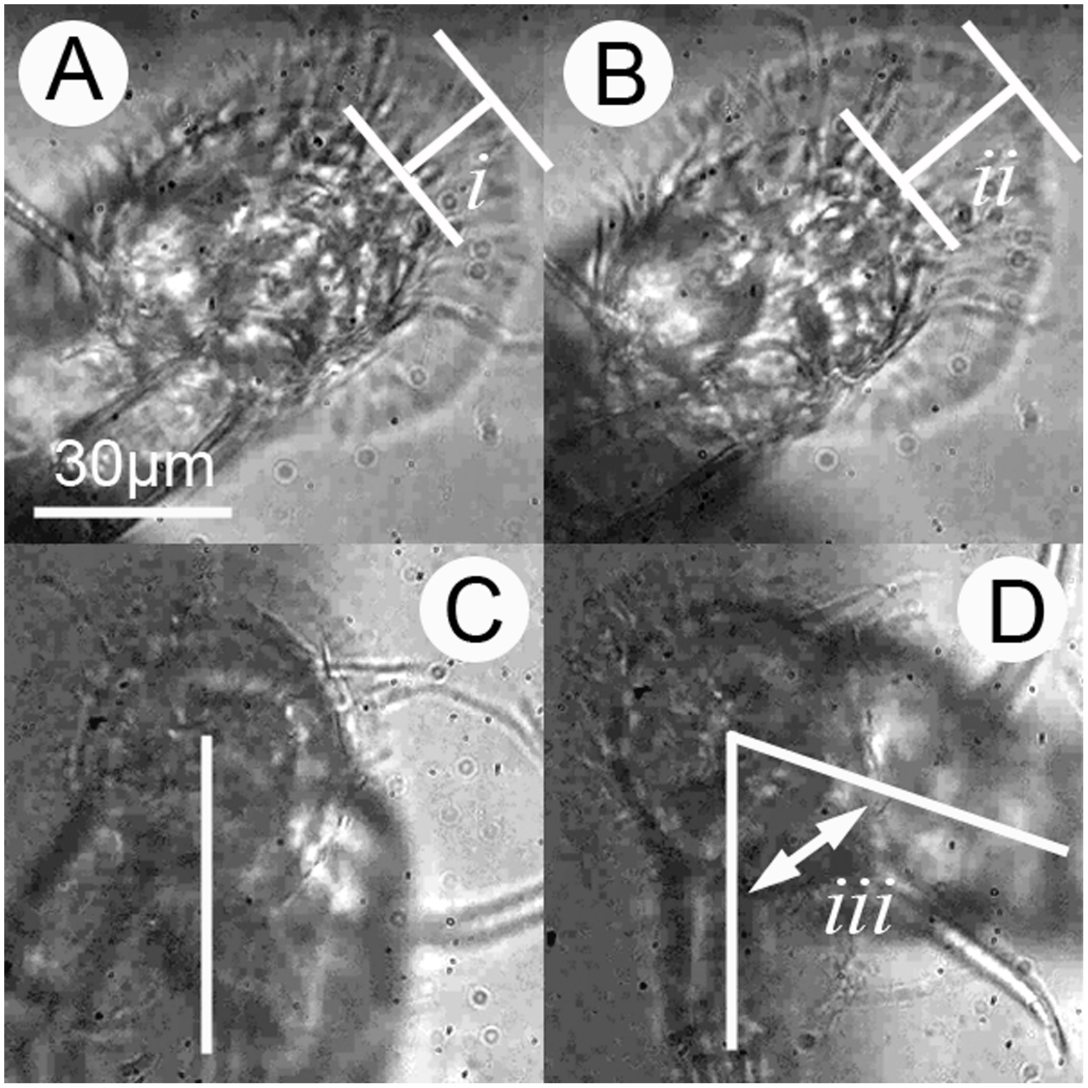
Illustration of changes at an adhesive interface under stress. A) The cyprid antennule viewed from below, with adhesive disc attached to the polystyrene surface and cuticular villi (i) in the resting state. B) As stress is applied to the system, the cuticular villi elongate and draw parallel to the surface (ii). When torsion forces are experienced, the antennule twists around the pivot point of the attached disc C) before detachment is initiated D) at around 70° from the resting position (iii).

The soft, malleable nature of the antennular disc surface and the velum encircling the 3^rd^ segment (Figure 1) was clear from the captured movies and likely serves three purposes: First, as described above, this flexibility allows the disc to be pushed against the surface, spreading out and maximising adhesion. Secondly, in a related adaptation, the flexible surface of the 3rd segment would allow conformation of the attachment disc to uneven surface textures, although this was not observed directly. In this capacity, the cuticular villi that cover the surface of the attachment disc (Figures 1 & 2) and the encircling velum are also crucial. Finally, a combination of the inherent flexibility of the limb and the presence of the cuticular villi at the adhesive interface endow the system with considerable resistance to lateral shear and torsion forces, as would be experienced in hydrodynamic flow and during close inspection of surfaces. With regard to the former, stress-induced peeling at the leading edge of the attachment disc was initiated only after 30% (±8.1 SD, n = 3) lateral displacement (equivalent to lap-shear) of the 3rd segment in the face of flow (Figure 2; Movie S4). During stress application, the villi drew parallel with the surface from their original upright position. The villi alone, therefore, provide a mechanical buffer between the surface and the bulk of the 3rd segment that absorbs applied force, graduate the change in modulus from the surface to the cyprid limb and, as a result, reduce stress concentration, lateral slip, tearing and peeling at the adhesive interface.

As well as resisting direct lateral forces, the villi also reduce interfacial slip as a result of torsion forces. Figure 2 (Movie S5) demonstrates an instance in which an antennule rotated 70° around the pivot point of the attachment disc before peeling was initiated at the adhesive interface. The disc villi and radial disc setae (see [5]) provide good reference for observing this behaviour, initially radiating orthogonally from the centre of the 3rd segment with their angle of incidence becoming more acute as twisting progresses.

The dynamicity of the disc surface, as seen in the supplementary movies, probably explains why the surface contour of the adhesive disc varies between individual specimens observed in SEM preparations. SEM micrographs indicated little if any variability in the density of villi over the entire disc surface in *S. balanoides* (Figure 1), except for immediately around the axial disc seta (see also [5]). This void correlates well with the pattern of temporary adhesive deposition observed by Phang et al. (2008) [18]. The average area of an adhesive disc was 477.72 µm^2^±54.2 (SD). Villus density was estimated to be 10.2 villi µm^2^±1.48 (SD) and did not vary between individuals (ANOVA F = 2.63 P = 0.085). Visible villus length was estimated to be 3.2 µm ±0.34 (SD) (see Figure 1C). By comparison with Moyse et al. [7], villus density in *S. balanoides* cyprids exceeded that in species that do not inhabit the rocky intertidal zone, perhaps indicating that density of villi could be correlated to substratum preference as seems to be the case for the shape of the entire third segment [33]. Under SEM conditions, the flaps of the velum were always closely applied to the sides of the 3rd segment, but this did not appear to be the case in living larvae. Further clarification is therefore required as to the exact role of the velum.

The only forces that the cuticular villi are not protected against are those generated by ‘lift’ in the normal direction since, once spread on the surface, the increase in attachment area they confer seems to be necessary to maintain strong adhesion. In this case, the highly extensible cuticle in the 2nd/3rd antennular joint probably confers some advantage, acting as a shock absorber [25].

It is unclear whether a truly ‘voluntary’ detachment mechanism exists for removal of a securely attached antennule from the surface. In some cases it appeared that cyprids used leverage to initiate peeling at the anterior or posterior fringe of the attachment disc, by drawing the 2nd antennular segment parallel to the surface and towards the body. It is possible, however, that different behaviours are exhibited depending on the activity of the cyprid. During rapid walking/wide exploration, as described earlier, full contact of the disc on the surface was rarely observed and instantaneous attachment/detachment occurred easily. Constant rolling of the 3rd segment during exploration (Movie S3) probably served a dual function; preventing more permanent attachment when not required and allowing intermittent ‘testing’ of the surface by the chemosensory axial and radial disc setae [5]. Cyprids probably also use a characteristic surface-bumping behaviour (Movies S6 & S7) for the same purpose. The latter behaviour further supports the observation that temporary adhesion is voluntary, and not an inevitable result of contact between the antennular discs and the surface.

Experience suggests that cyprids engaged in wide search behaviour are also more easily detached by instantaneous forces than those engaging in close searching or inspection, suggesting that spreading of the attachment disc on the surface may be necessary to secure a more tenacious grip. For cyprids that spread the disc on the surface and, presumably, attached more tenaciously, the forceful tugging observed during the natural detachment process did not appear consistent with the ability of cyprids to ‘voluntarily’ detach. Again, Crisp [26] first hypothesised that this behaviour could supply information to the cyprid relating to adhesive tenacity and thereby influence the likelihood of settlement to a specific surface. In most cases, this tugging ultimately initiated peeling at the periphery of the disc and subsequent adhesive failure in a 2-phase manner. The first, most energetic phase, involved breaking the full disc bond and resulted in a partial attachment similar to that used in wide searching behaviour – often cyprids remained adhered just by the very periphery of the disc (Movie S8), which was enough to maintain surface attachment in the absence of flow. The second phase was complete removal of the disc from the surface, which was often accompanied by a ‘jerking’ motion signifying the release of stored energy in the system (Movie S9). There seemed to be no mechanism to allow low-effort release from the surface following disc spreading.

For securely attached cyprids, detachment also seemed to be time dependant. In a few cases, the velum and peripheral fringe of the attachment disc, that is brought into contact with the surface during disc spreading, apparently peeled away from the surface spontaneously after a period of time as if ‘spring-loaded’, initiating detachment. At this point, spreading could be re-established if required or the cyprid could effortlessly remove the antennular disc from the surface. Unfortunately it was not possible to quantify the time period after which this process occurred using the present techniques. In all cases, ‘voluntary’ detachment of cyprids from the surface was a result of peeling of the disc that could be measured in tenths of seconds.

### Footprint Deposition

**Figure 3:**
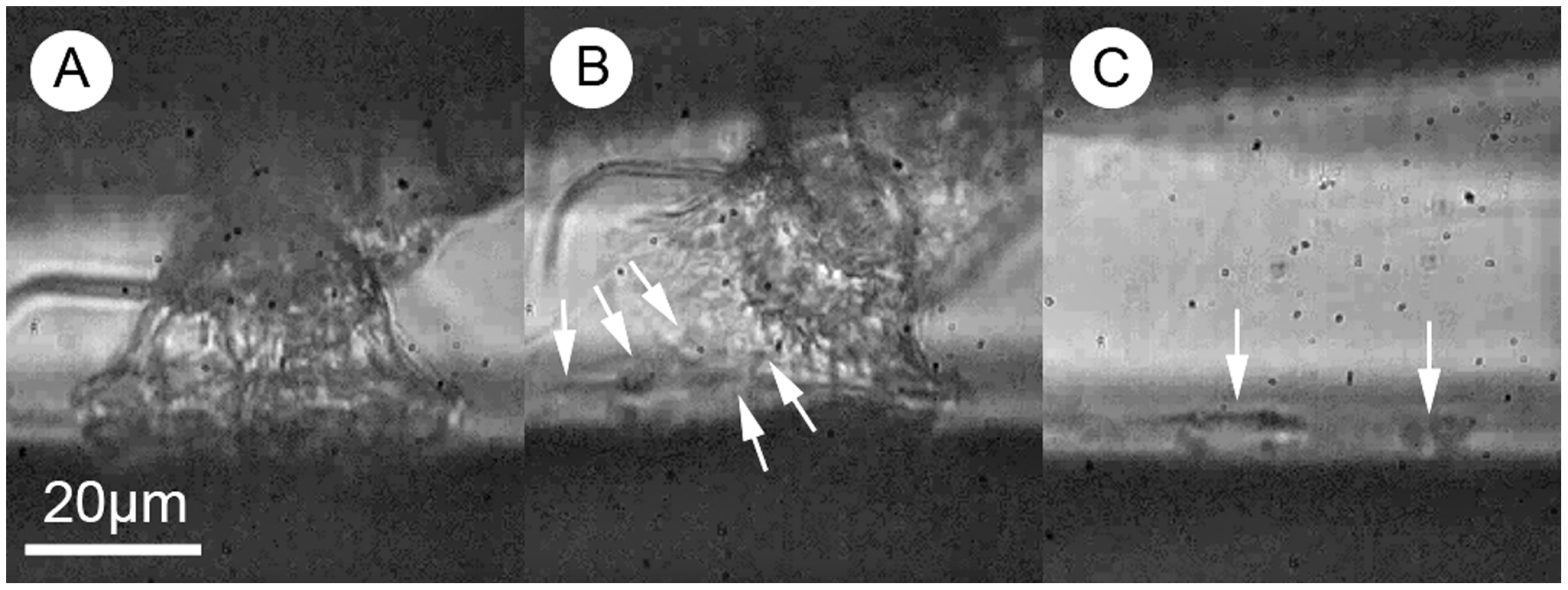
Deposition of a footprint on a surface by an exploring cyprid. A) Temporary attachment of the antennular disc to the surface. B) Peeling of the antennular disc from the surface (white arrows indicate stretching of a viscoelastic material between the disc and the surface – presented more clearly in Movie S10). C) Deposited footprint on the surface as the cyprid moves away.

Very occasionally, during peeling of the disc from the surface, it was possible to observe the deposition of a temporary adhesive ‘footprint’ (Movie S10; Figure 3). The composition and even the exact role of the temporary adhesive are currently unclear, although it has been demonstrated experimentally that footprints of this material deposited onto surfaces contain a large cuticular glycoprotein known as the barnacle settlement-inducing protein complex (SIPC; [17], [29]) and act as a conspecific cue to settlement [16], [27]. It is presumed that the temporary adhesive is also important in the temporary adhesion of cyprids to surfaces, however its macroscopic properties have never been investigated. Previously, the only studies to visualise cyprid footprint deposition *in situ* were Andersson et al. [21] and Aldred et al. [22], who both used low-magnification imaging surface plasmon resonance to visualise the footprints. Although this technique proved useful in quantitatively comparing surfaces on the basis of their affinity for cyprid footprints, it was not possible to observe the biomechanical nature of the material itself.

If the footprint material were the principle mechanism of adhesion in this system, as was the prevailing view throughout the 1980s–90s, then it would belong to the category of so called ‘wet’ adhesives [34]. Wet adhesives do not ‘cure’ or harden over time and may be divided broadly into those relying on either viscous or viscoelastic mechanisms of action. In ideal circumstances, a purely viscous material will not only resist detachment as a function of its viscosity, but also maintain adhesion between two solid surfaces in a fluid medium by capillary action [9]. In reality, however, micro-scale systems such as the cyprid antennule are likely dominated by the viscosity of the ‘adhesive’ rather than any resulting capillary forces. The viscous/capillary mechanism has been cited previously as a possible candidate to explain cyprid temporary adhesion [26], although there was some ambiguity regarding the author’s use of the term ‘Stéfan adhesion’ which implied a different mechanism. Capillarity is an attractive process in a three-phase system whereby a spreading liquid, separate to the surrounding medium, draws two solid surfaces together. Stéfan adhesion on the other hand is a resistance to viscous flow in a two-phase system and does not, in itself, imply attraction.

Capillary action alone can certainly provide strong adhesion, however despite being very resistant to tensile stress, systems that rely exclusively on capillarity are highly susceptible to slip in the shear plane. As demonstrated in Figure 2 A & B, cyprids do not suffer from this weakness and their adhesion is therefore not simply a capillary mechanism - although capillarity could of course be involved. On the basis of this evidence the temporary adhesion of cyprids must involve a viscoelastic wet adhesive; a ‘sticky’ adhesive in the conventional sense with strong affinity for diverse surfaces underwater.

From the video analysis (Movie S10), it appears that the footprint material is indeed a rather sticky, viscoelastic substance. Phang et al. [20] used atomic force microscopy (AFM) to characterise nanoscopically the mechanical behaviour of this material, reporting an absence of strain-rate dependence in the force required to rupture protein aggregates, therefore implying viscous, rather than viscoelastic character for subunits of the footprint material. However, caution must always be exercised when extrapolating such data to the whole-organism and it is clear that in its natural application the footprint material is highly elastic and not simply viscous (Movie S10). In fact, when testing droplets of spider adhesive, Sahni et al. [35] only observed viscoelastic character at strain rates above 10 µm/second, whereas Phang et al. [20] performed experiments between 0.5 and 11 µm/second. It is perhaps not surprising, therefore, that only a gradual increase in rupture force was observed in the range of strain rates tested by Phang et al. [20]. It should also be noted that the strain rates used by Phang et al. were, in all likelihood, far below those that would be relevant to this adhesive system in the natural environment.

So, from the behaviour of cyprids during attachment, the *in vivo* appearance of the attachment disc at high magnification and the apparently sticky, viscoelastic nature of the footprint secretion, it is now possible to state with some confidence that the original definition of this material was probably accurate [15]. The proteinaceous substance secreted onto the attachment disc during surface exploration by cyprids is not *simply* a conspecific cue or water excluder to facilitate dry adhesion by the cuticular villi (although it may still fulfil these functions of course; see [9] for discussion), rather the available evidence strongly supports an adhesive function.

### Involuntary Detachment from Surfaces

Different lineages of invertebrates have independently evolved sophisticated mechanisms for resisting involuntary detachment from surfaces. Echinoderm temporary adhesion is, to all intents and purposes, a permanent bond until a de-adhesive is secreted by the organism to initiate release [30]. Although this ‘duo-gland’ mechanism has been postulated for temporary adhesion in cyprids [26], no evidence has ever been offered in support of it and the observations presented here would also suggest that it is an unlikely explanation. Some arthropods, for example stick insects, use oil/water emulsions as non-Newtonian fluids to increase the resistance of their viscous adhesive system to lateral slip [36]. Again the cyprid footprint appears, at least in physicochemical terms, to be a homogeneous material [19] and it seems more likely that a combination of the cuticular villi and the viscoelastic nature of the cyprid temporary adhesive fulfil this function adequately.

**Figure 4:**
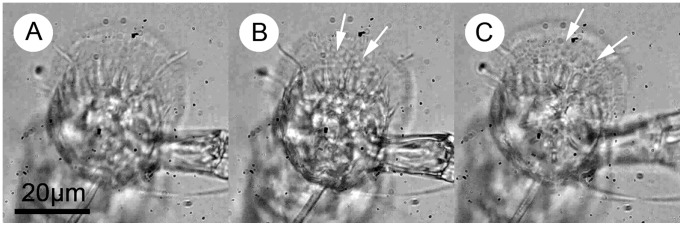
Formation of putative microbubbles at the adhesive interface during forced detachment of a cyprid adhesive disc from a polystyrene surface, viewed from beneath. The phenomenon is presented more clearly in Movie S11. A) The antennular disc attached normally to the surface during inspection behaviour. B) Application of force initiates the formation of bubbles at the interface, indicated with white arrows. C) Progressively increasing force leads to detachment. Bubbles still visible at the instant of detachment (white arrows).

Cyprid temporary adhesion does, however, benefit from one mechanism that seems not to have been reported to date in the bioadhesives of marine organisms. During experiments in which cyprids were removed from the surface by force, putative microbubbles were observed to form spontaneously at the adhesive interface at the instant of removal (Figure 4; Movie S11). Most good synthetic adhesives of the ‘tacky’ variety are soft, viscoelastic and highly deformable, often stretching up to 10 times their original thickness under tension [37]. These adhesives are designed so that, in the initial stages of tensile fracture, microbubbles are free to form at the adhesive/surface interface and rapidly proliferate in the normal direction with increasing strain (or z-deformation). This process quickly turns the 2-dimensional adhesive film into a 3-dimensional foam [38] dissipating large amounts of energy as it does so. The bubbling that occurs in soft adhesive films during separation is often referred to as ‘cavitation’, although the alternative term ‘crazing’ is preferred here. Strictly speaking, the process referred to in the present context does not conform to either definition. Cavitation, although it can be an attractive force [39], usually describes a first order phase change (i.e. liquid to gas) that occurs as a result of localised, instantaneous pressure reduction in a fluid system. This leads to bubble formation and dissipation of large quantities of energy, but it is not the mechanism at work in wet adhesive joints. Crazing on the other hand does not refer specifically to a phase change of the material, but rather a mechanical property that allows formation of cavities at stress points associated with flaws in the material or stress nucleation sites (in this case, the cuticular villi of the attachment disc). Crazing absorbs fracture energy under strain and increases the fracture toughness of a material immensely. Since many individual crazes nucleate at different points throughout the material, crazing is also typified by homogeneous distribution of force throughout the material or bond, delaying the formation of stress singularities and further increasing the resistance of the material to failure. This phenomenon is, however, typically associated with brittle solid materials rather than adhesive gels like the cyprid temporary adhesive. In any case, materials that form cavities under strain in this manner, regardless of nomenclature, dissipate much of the energy applied prior to separation and therefore show enhanced adhesive qualities.

Based on the information above, the temporary adhesion of barnacle cyprids will be defined here as ‘soft adhesion’ - use of a viscous, mucous-like glue that does not harden with time (See [18]–[20] for cyprid-specific data), as opposed to ‘hard adhesion’ that would involve an adhesive with progressive curing, such as that of the adult barnacle. So called ‘hard’ and ‘soft’ adhesion mechanisms respond very differently to tensile and shear stress, and the different behaviours of the two systems have implications not only for adhesion strength testing of fouling organisms, such as barnacles, but also for the development of bio-inspired adhesives for niche applications. Unfortunately for the purposes of simple interpretation, most biological adhesion systems fall into the ‘soft’ category [40], whereas most mechanical theory and testing relates to ‘hard’ adhesion. Because these systems are designed for different applications and respond differently to stress, caution must be exercised when presuming rigidity in a system, even for calcified macroorganisms such as adult barnacles [41].

One consideration for future mechanical characterisation of cyprid temporary adhesion is that peak force measurements such as those used by [13], although relevant in the context of their study, may be unrepresentative of the real work required to break adhesive bonds in soft/deformable systems and novel methods must be developed to build on their observations. In soft systems, especially soft viscoelastic systems, it is not unusual for an adhesive bond with a low peak removal force to require greater total energy input for rupture than a bond that manifests a higher peak removal force for the same strain rate. Indeed, this is one of the strengths of ‘soft adhesion’. To the tester with an interest in biofouling, for example, this distinction may be irrelevant since the available energy input is effectively infinite. From a biological perspective however, it is highly relevant as the forces likely to remove cyprids from surfaces in the natural environment (sudden hydrodynamic effects, impact forces and/or predators) are likely to occur only for a fixed duration and have finite energy. In this context, mechanisms for dissipating energy and delaying the application of peak force, especially for a temporary adhesion mechanism, may be more advantageous than increasing the ultimate tensile strength of the adhesive bond. Rather than using ultimate tensile stress to describe biological adhesion, therefore, work of adhesion (the total effort required to separate two adhered materials) is instead considered to be the more relevant measure for soft/temporary adhesion systems in a natural context. The putative crazing of the cyprid temporary adhesive is a good example of such an energy dispersive process and suggests that previous interpretations of the effectiveness of the cyprid temporary adhesion mechanism may have been considerable underestimates.

In morphological terms, the crazing of the temporary adhesive probably also explains the characteristically ‘rough’ appearance of cyprid footprints at the nanoscale [18]–[19]. The larger voids present on micrographs of footprints may demark the locations of larger cavity formation, whereas the smaller voids, which correlate to the villi on the disc surface [18], may be nucleation sites of smaller cavities.

### Conclusions

Previously undescribed behaviours have been identified during wide searching, inspection and detachment by exploring cyprids that tailor temporary adhesion specifically to the requirements of each exploration stage. The attachment disc of the 3rd antennular segment was only rarely in full contact with the surface during wide searching, ensuring relatively weak attachment. For the more secure attachment required during inspection, the disc was brought into full contact and spread out on the surface. Removal of the attachment disc from the surface was not simply a reversal of the attachment process. Detachment during wide searching seemed to be facilitated for the most part by minimising the time that the disc was in full contact with the surface. Following the more secure attachment used during inspection, truly ‘voluntary’ detachment was never observed. Rather, detachment seemed to be facilitated either by progressive reduction in bond strength over time, or by ‘brute-force’, with the cyprid tugging on the antennule until rupture was initiated. From an evolutionary perspective, development of a temporary adhesive mechanism without a mode of detachment is somewhat unsatisfactory, but it certainly seems that in this case the voluntary element is to either minimise or maximise adhesion strength, depending on circumstance.

During ‘involuntary’ detachment by force, micro bubbles were observed at the adhesive-surface interface and it is suggested that these allow dissipation of energy in the system prior to adhesive failure. They may also explain the characteristic morphology of cyprid footprints imaged using AFM. General observation of the footprint material and the behaviour of the adhesive system under stress suggest that it does, indeed, function as a true adhesive and is therefore a promising target for the development of synthetic bio-inspired glues and future marine antifouling strategies [42].

## Supporting Information

Movie S1 The attachment of an antennule to a surface during the first phase of an exploratory footstep. Note the flexibility and degree of spreading of the antennular disc on the surface (sequence ∼1 s).
(AVI)

Movie S2 The detachment of an antennule from a surface during the second phase of an exploratory footstep (sequence ∼1 s).
(AVI)

Movie S3 Rolling movement of the antennular disc. If a cyprid remains in one spot for longer than the momentary full contact mid-stride, the disc is ‘rolled’ on the surface. This behaviour has putative dual role, serving a sensory function as well as minimising adhesion to the surface (sequence ∼1 s).
(AVI)

Movie S4 Compliance of the 3rd antennular segment in the shear direction. As detachment force is applied by the cyprid, the joint between the 2nd and 3rd antennular segments, visible through the 3rd segment, can be seen to move laterally relative to the area of attachment disc in contact with the surface. As this occurs, the velum, radial disc setae and cuticular villi can be seen to elongate. Finally, the adhesive bond is broken at the leading fringe of the disc (sequence ∼2 s).
(AVI)

Movie S5 Rotation of the 2nd antennular segment by ∼70° around the attachment point. The velum and radial disc setae can be seen twisting during this process, but retain their original points of contact with the surface until detachment (sequence ∼2 s).
(AVI)

Movie S6 A cyprid of *Semibalanus balanoides* engaging in characteristic ‘bumping’ behaviour on a surface. This behaviour has a putative sensory role and also demonstrates that there can be contact of the adhesive discs and the surface without adhesion occurring.
(AVI)

Movie S7 A higher magnification sequence of the behaviour in Movie S6.
(AVI)

Movie S8 Significant adhesion force can be maintained simply by contact of the periphery of the attachment disc with the surface.
(AVI)

Movie S9 A different view of the type of attachment behaviour in Movie S8, demonstrating the ‘jerking’ movement accompanying removal of the adhesive disc from the surface, implying adhesion.
(AVI)

Movie S10 Deposition of cyprid temporary adhesive to a surface during exploration. The adhesive material can be seen as a viscoelastic fluid that bridges the interface between the surface and the disc briefly before springing to the surface (sequence ∼1 s).
(AVI)

Movie S11 The formation of putative microbubbles at the interface between the antennular disc/temporary adhesive and the surface during removal under strain. The impression of these bubbles seems to remain on the disc surface momentarily following removal (sequence <1 s).
(AVI)
